# The impact of the Affordable Care Act on health care access and self‐assessed health in the Trump Era (2017‐2018)

**DOI:** 10.1111/1475-6773.13549

**Published:** 2020-08-31

**Authors:** Charles Courtemanche, James Marton, Benjamin Ukert, Aaron Yelowitz, Daniela Zapata

**Affiliations:** ^1^ Department of Economics Gatton College of Business and Economics University of Kentucky Lexington Kentucky; ^2^ National Bureau of Economic Research Cambridge Massachusetts; ^3^ Institute of Labor Economics (IZA) Bonn Germany; ^4^ Department of Economics Andrew Young School of Policy Studies Georgia State University Atlanta Georgia; ^5^ Department of Health Policy and Management Texas A&M University College Station Texas; ^6^ Impaq International Washington DC USA

**Keywords:** access to care, affordable care act, health, health care access, health insurance, medicaid expansions, self‐assessed health, self‐reported health

## Abstract

**Objective:**

To estimate the impact of the major components of the ACA (Medicaid expansion, subsidized Marketplace plans, and insurance market reforms) on health care access and self‐assessed health during the first 2 years of the Trump administration (2017 and 2018).

**Data Source:**

The 2011‐2018 waves of the Behavioral Risk Factor Surveillance System (BRFSS), with the sample restricted to nonelderly adults. The BRFSS is a commonly used data source in the ACA literature due to its large number of questions related to access and self‐assessed health. In addition, it is large enough to precisely estimate the effects of state policy interventions, with over 300 000 observations per year.

**Design:**

We estimate difference‐in‐difference‐in‐differences (DDD) models to separately identify the effects of the private and Medicaid expansion portions of the ACA using an identification strategy initially developed in Courtemanche et al (2017). The differences come from: (a) time, (b) state Medicaid expansion status, and (c) local area pre‐2014 uninsured rates. We examine ten outcome variables, including four measures of access and six measures of self‐assessed health. We also examine differences by income and race/ethnicity.

**Principal Findings:**

Despite changes in ACA administration and the political debate surrounding the ACA during 2017 and 2018, including these fourth and fifth years of postreform data suggests continued gains in coverage. In addition, the improvements in reported excellent health that emerged with a lag after ACA implementation continued during 2017 and 2018.

**Conclusions:**

While gains in access and self‐assessed health continued in the first 2 years of the Trump administration, the ongoing debate at both the federal and state level surrounding the future of the ACA suggests the need to continue monitoring how the law impacts these and many other important outcomes over time.

**What is already known on this topic**

- The ACA led to significant improvements in coverage and access to care throughout 2014 to 2016, as well as a lagged emergence of improvements in self‐assessed health.
- However, changes in ACA administration beginning in 2017 could have negatively affect these gains.
- This study evaluates the impact of the ACA on insurance coverage, access to care, and self‐assessed health including 2017 and newly released 2018 data.

**What this study adds**

- Despite a political shift and changes in the administration of the ACA beginning in 2017, gains in coverage and access to care remained stable in 2017 and 2018 compared to 2016.
- In addition, we also continue to observe improvements in excellent self‐assessed health in 2017 and 2018.

## 1 INTRODUCTION

In 2014, the major components of the Affordable Care Act (ACA), including the individual mandate, subsidized Marketplace coverage, and state Medicaid expansions, were implemented.^1^, ^2^, ^3^ A recently published review^2^ summarizes the growing literature on the impact of the ACA on insurance coverage,^4^, ^5^, ^6^, ^7^, ^8^, ^9^, ^10^, ^11^, ^12^, ^13^, ^14^, ^15^, ^16^ access to care,^17^, ^18^, ^19^ and self‐assessed health^3^, ^20^, ^21^, ^22^, ^23^, ^24^, ^25^, ^26^, ^27^, ^28^ among other outcomes. This review suggests that the ACA, including the Medicaid expansions, increased coverage and access to care after one (2014) to four (2014‐2017) postreform years, but did not have as clear an effect on self‐assessed health. Some studies focusing on the ACA Medicaid expansions alone found mixed results, with some showing improvements in health, while others found no effect.^2^, ^3^ One study examining both the Medicaid expansions and the non‐Medicaid expansion components of the ACA found that an increase in health (as measured by the probability of reporting excellent health) emerged with a lag in 2015.^28^

In this paper, we estimate the causal effects of the ACA on access to care and self‐assessed health during 2017 and 2018, the first 2 years of the Trump administration, using data from the Behavioral Risk Factor Surveillance System (BRFSS). Ours is among the first publications to include both 2017 and 2018 data.^25^, ^27^, ^28^, ^29^, ^30^, ^31^ Our access outcomes are the likelihoods of having insurance coverage, costs being a barrier to seeking care, a primary care doctor, and a checkup in the past year. Our health outcomes include overall self‐assessed health and days of the previous month not in good physical health, not in good mental health, and with health‐related limitations.

There are multiple reasons why adding data from the first two years of the Trump administration to the prior analysis is important. First, even in the absence of any changes to the ACA, it would be interesting to see whether the lagged emergence of improved health continues into 2017 and 2018. This may be the case as enrollees become more familiar with their new coverage over time and how to best navigate the health care system. In addition, if upfront investments in medical care take time to translate into better health, then we might expect further improvement in self‐assessed health in 2017 and 2018.^32^ Second, 2017 marked a change in the administration of the ACA with a new president taking office in January. President Trump's first executive order encouraged the federal government to waive or delay the implementation of any components of the ACA that would impose a financial or regulatory burden.^33^ In addition, funding for ACA outreach and education programs, including funding for navigators, was reduced for open enrollment periods associated with 2017 and 2018 coverage.^34^ Potentially most consequential, in October 2017, the administration discontinued cost‐sharing reduction (CSR) payments to insurers for silver Marketplace plans, at a time when insurers already submitted premium rates for the coming plan year with an expectation of receiving CSR payments in return for reducing the cost‐sharing in plans for low income enrollees.^35^ Political debate surrounding the ACA was prominently featured in the news, including the failed vote to repeal the ACA in July 2017 and the vote to pass the tax reform package that included a repeal of the ACA individual coverage mandate in December 2018.^36^ Thus, the addition of 2017 and 2018 data allows us to examine the initial causal impact of these events. This is important as recent descriptive evidence suggests that the national coverage rate actually fell by 0.5 percentage points between 2017 and 2018.^37^

Following a recently established literature, we estimate difference‐in‐difference‐in‐differences (DDD) models with the differences coming from time, state Medicaid expansion status, and local area pretreatment uninsured rate in order to estimate the impact of the full ACA.^11^, ^14^, ^15^, ^16^, ^25^, ^27^, ^28^, ^38^ This approach stands in contrast to many studies that use a simpler difference‐in‐differences (DD) model comparing changes in expansion states to changes in nonexpansion states in order to identify the effect of the ACA Medicaid expansion alone. Identifying the impact of the national components of the ACA, such as the individual mandate and subsidized Marketplace coverage, requires a different approach because they were implemented in every state at the same time. The inclusion of a third difference in our model handles this issue because the national components of the ACA should provide the most intense “treatment” in local areas with the highest uninsured rates prior to the ACA.

## 2 DATA AND METHODS

### 2.1 Data

We use data from the BRFSS, an annual telephone survey of health and health behaviors conducted by state health departments in collaboration with the CDC. The BRFSS is the largest continuous health survey in the United States, collecting information on more than 300 000 adults per year. Having a large sample size is critical to obtaining meaningful precision because the ACA affected insurance coverage for only a fraction of the population, limiting plausible effect sizes. The BRFSS is therefore a commonly used data source in the ACA literature on access and self‐assessed health.^23^, ^25^, ^27^, ^28^

Our sample period is 2011‐2018. The sample starts in 2011 because this is the first year in which the BRFSS included cell phones in its sampling frame. The sample ends in 2018 because this is the last year currently available. This timeframe gives us three years of pretreatment data and five years of post‐treatment data. We limit our sample to individuals 19‐ to 64 years old who were interviewed between 2011 and 2018. As is common in the literature, we drop observations with missing values for the variables used in our analysis.^23^, ^39^, ^40^

Our outcome variables measure access to care and self‐reported health status. Access outcomes include indicators for any health coverage, having a primary care doctor, having a regular physician checkup in the past 12 months, and having any care needed but foregone because of cost in the past 12 months. Self‐reported health status is based on a rating of overall health as poor, fair, good, very good, or excellent. We use this to construct indictors for whether overall health is good or better (ie, good, very good, or excellent), very good or excellent, and excellent. Other health measures include number of days of the last 30 not in good mental health, not in good physical health, and with health‐related functional limitations. These sorts of subjective self‐assessed health variables have been shown to be correlated with objective measures of health, such as mortality.^41^, ^42^, ^43^

We construct a Medicaid expansion indicator that is based on information collected by the Kaiser Family Foundation.^44^ A total of 31 states and Washington, DC expanded Medicaid by 2016 and no state expanded in 2017 or 2018. The majority of states expanded Medicaid in January 2014, with some exceptions. Michigan expanded in April 2014 and New Hampshire in August 2014. Pennsylvania, Indiana, and Alaska expanded in January, February, and September of 2015, respectively. Montana and Louisiana expanded in January and July of 2016, respectively. We classify states as part of the Medicaid expansion beginning the month‐year of their expansion. Other state‐level variables include indicators for whether states set up their own insurance exchanges, whether these exchanges experienced glitches,^44^, ^45^ and seasonally adjusted monthly state unemployment rate from the Bureau of Labor Statistics. The exchange glitch indicator flags the six states that had severe upfront technology problems when they rolled out their state exchange in 2014.^45^

We measure the intensity of the non‐Medicaid components of the ACA using the uninsured rate in the respondent's “local area” in the pretreatment year of 2013. This measure captures the “dose” of ACA treatment the local area could have received. We compute each respondent's “local area” pretreatment uninsured rate within our BRFSS sample of nonelderly adults. The publicly available BRFSS does not include geographic identifiers narrower than the state, but does tell us whether the respondent resides in the center city of an MSA, outside the center city of an MSA but inside the county containing the center city, inside a suburban county of an MSA, or not in an MSA. We use this variable to construct four subgroups within each state: those living within a central city, suburbs, non‐MSA, and within‐state location unavailable (this is the case for respondents interviewed on their cell phone). Based on these four geographic categories, we calculate the pretreatment average uninsured rates by “location” (considering “cell phone” to be a location for the sake of convenience) within a state. To ensure that each area contains enough respondents from our sample to reliably compute pretreatment uninsured rates, we follow the previous literature and combine the seven areas with fewer than 200 respondents in 2013 with other larger areas. Specifically, we combine the central city and suburban parts of Wyoming into one area, and do the same for Vermont, South Dakota, and Montana. In addition, we combine the suburban and rural parts of the states of Massachusetts, Arizona, and California. This process, which exactly mirrors that used in prior BRFSS studies of the ACA’s two‐ and three‐year effects,^25^, ^27^ generates 194 areas with 2013 uninsured rates that are computed from 219 to 5,804 respondents, with the average being 1,475 respondents and the median being 1,205.

We use responses from several other BRFSS questions to construct individual‐level controls. Specifically, we control for age using indicators for five‐year increments (from 25‐29 to 60‐64, with 19‐24 as the reference group), female, race/ethnicity (non‐Hispanic black, Hispanic, and other; non‐Hispanic white as the reference), married, education (high school degree, some college, and college graduate; less than high school degree as the reference), household income ($10 000‐$15 000, $15 000‐$20 000, $20 000‐$25 000, $25 000‐$35 000, $35 000 $50 000, $50 000‐$75 000, and >$75 000, with <$10 000 as the reference), indicators for the number of children in the household, whether primary occupation is student, and whether the respondent is unemployed.

Table 1 provides pretreatment means and standard deviations of our ten outcomes of interest between 2011 and 2013, and Appendix Table S1 reports the means and standard deviations for the controls. We stratified our entire analytic sample into four groups based on whether the respondent's state expanded Medicaid and whether the local area's pretreatment uninsured rate was above or below the median within the sample. According to Table 1, 79 percent of the sample had some form of coverage prior to 2014. Individuals in expansion states (columns 2 and 3) were slightly more likely to have insurance prior to 2014 than those in nonexpansion states (columns 4 and 5). Residents who live in expansion states with prereform uninsured rates below the median (column 3) had, on average, better health care access and self‐assessed health than the rest of the sample even before 2014. Our DDD model will account for these baseline differences. Our online Appendix describes trends in our outcome variables over time.

**TABLE 1:** Means and standard deviations of dependent variables by state medicaid expansion status and pretreatment uninsured rate

	Full sample	Medicaid expansion; ≥Median baseline uninsured	Medicaid expansion; <Median baseline uninsured	Nonexpansion; ≥Median baseline uninsured	Nonexpansion; <Median baseline uninsured
Any insurance coverage	0.788 (0.409)	0.732 (0.443)	0.855 (0.352)	0.686 (0.464)	0.831 (0.375)
Primary care doctor	0.741 (0.439)	0.650 (0.477)	0.816 (0.386)	0.634 (0.482)	0.814 (0.392)
Checkup	0.627 (0.234)	0.559 (0.497)	0.660 (0.473)	0.592 (0.491)	0.680 (0.467)
Cost barrier to care in past year	0.192 (0.394)	0.232 (0.421)	0.147 (0.125)	0.256 (0.436)	0.170 (0.376)
Overall health good or better	0.840 (0.367)	0.830 (0.376)	0.852 (0.355)	0.826 (0.379)	0.842 (0.363)
Overall health very good or better	0.536 (0.499)	0.518 (0.499)	0.559 (0.497)	0.506 (0.499)	0.544 (0.498)
Overall health excellent	0.204 (0.403)	0.200 (0.400)	0.209 (0.407)	0.200 (0.400)	0.197 (0.399)
Days not in good physical health in past month	3.648 (7.964)	3.738 (7.986)	3.547 (7.792)	3.630 (7.992)	3.807 (8.231)
Days not in good mental health in past month	4.108 (8.210)	4.560 (8.510)	3.864 (7.907)	4.269 (8.432)	3.905 (8.130)
Days with health‐related limitations in past month	2.508 (6.779)	2.596 (6.808)	2.416 (6.647)	2.532 (6.849)	2.590 (6.999)

Note

Standard deviations in parentheses.

### 2.2 Methods

Our goal is to estimate the effects of both the fully implemented ACA (including the Medicaid expansion) and the ACA without the Medicaid expansion for each of our ten access and health outcomes. Most previous studies in the ACA literature estimated only the effect of the Medicaid expansion using a difference‐in‐differences (DD) approach. Our goal is more ambitious in that we aim to also identify the causal effect of the ACA’s national treatment (ie, the package of reforms related mostly to private insurance), which creates the challenge of disentangling its impact from underlying year‐to‐year fluctuations in our outcomes that would have occurred even in the absence of the ACA. To address this challenge, we adopt a difference‐in‐difference‐in‐differences (DDD) strategy used in several recent ACA studies that also aimed to separately identify the effects of the national and Medicaid portions of the law. The DDD strategy utilizes “differences” coming from time, state Medicaid expansion status, and local area pretreatment uninsured rate, with the central idea being that coverage expansions provide the most intense treatments in areas with high baseline uninsured rates.^11^, ^14^, ^15^, ^16^, ^25^, ^27^, ^28^, ^38^

Formally, the model with a simple combined five‐year post period (2014‐2018) is

(1) $$y_{\mathrm{iast}}=\gamma_{0}+\gamma_{1}\left({\mathrm{UNINSURED}}_{\mathrm{as}}*{\mathrm{POST}}_{t}\right)+\gamma_{2}{(\mathrm{MEDICAID}}_{\mathrm{st}}*{\mathrm{POST}}_{t})+\gamma_{3}\left({\mathrm{UNINSURED}}_{\mathrm{as}}*{\mathrm{MEDICAID}}_{s}*{\mathrm{POST}}_{t}\right)+\gamma_{4}X_{iast}+\theta_{\mathrm{at}}+\alpha_{\mathrm{as}}+\varepsilon_{\mathrm{iast}}$$

where

- $y_{\mathrm{iast}}$ is a generic outcome for individual *i* in area type (central city, rest of MSA, non‐MSA, cell phone) *a* in state *s* in month/year *t*,
- ${\mathrm{POST}}_{t}$ equals one in period *t* if it is in the postreform period of January 2014 or later,
- $X_{iast}$ is a vector of controls,
- ${\mathrm{MEDICAID}}_{s}$ indicates state participation in the ACA’s Medicaid expansion,
- ${\mathrm{UNINSURED}}_{\mathrm{as}}$ is the 2013 uninsured rate in area type *a* within state *s*,
- $\theta_{\mathrm{at}}$ denotes fixed effects for each time‐by‐area‐type combination (eg, central city in April 2011),
- $\alpha_{\mathrm{as}}$ denotes fixed effects for each area (eg, non‐MSA in Kentucky),
- and $\varepsilon_{\mathrm{iast}}$ is the error term, which is clustered by state and heteroscedasticity‐robust.

Note that ${\mathrm{POST}}_{t}$ is absorbed by the time‐by‐area‐type fixed effects ($\theta_{\mathrm{at}}$) so it is not separately included in Equation (1), while the terms ${\mathrm{UNINSURED}}_{\mathrm{as}}$, ${\mathrm{MEDICAID}}_{s}$, and ${\mathrm{UNINSURED}}_{\mathrm{as}}*{\mathrm{MEDICAID}}_{s}$ are absorbed by the area fixed effects ${(\alpha }_{\mathrm{as}}$). The time‐by‐area‐type fixed effects ($\theta_{\mathrm{at}}$) would subsume the inclusion of standard time fixed effects, which implies they control for national trends, such as changes in the national unemployment rate. We use sampling weights to account for the complex survey design.

In Equation (1), the effect of the national portion of the ACA alone is given by $\gamma_{1}$*${\mathrm{UNINSURED}}_{\mathrm{as}}$, which means it is assumed to be zero at a zero percent baseline uninsured rate and to increase linearly as this rate rises. This is meant to capture national aspects of the ACA outside of the Medicaid expansion, such as subsidized Marketplace coverage and the individual mandate. In addition to incentivizing purchase on the Marketplace, the mandate may have led to a “woodwork” effect where people previously eligible for coverage through their employer or Medicaid but not enrolled decided to go ahead and enroll. Similarly, the effect of the Medicaid expansion alone is given by $\gamma_{3}*{\mathrm{UNINSURED}}_{\mathrm{as}}*{\mathrm{MEDICAID}}_{s}$, meaning it is zero in nonexpansion states (where ${\mathrm{MEDICAID}}_{s}=0$) and $\gamma_{3}*{\mathrm{UNINSURED}}_{\mathrm{as}}$ in expansion states (where ${\mathrm{MEDICAID}}_{s}=1$). Following prior literature, we consider $\gamma_{2}$ to represent unobserved confounders rather than capturing part of the Medicaid expansion's causal effect, though we test the sensitivity of our results to changes in this assumption.^11^, ^14^, ^15^, ^16^, ^25^, ^27^, ^28^, ^38^, ^46^ The effect of the “fully implemented” ACA, that is, in Medicaid expansion states, combines the impacts of the Medicaid expansion and the national non‐Medicaid components of the ACA: $\gamma_{1}$*${\mathrm{UNINSURED}}_{\mathrm{as}}+\gamma_{3}*{\mathrm{UNINSURED}}_{\mathrm{as}}$. We report the predicted or implied effect of the ACA at the sample mean pretreatment uninsured rate rather than the underlying regression coefficients. These implied effects are given by $\gamma_{1}$*$\overset{‐}{{\mathrm{UNINSURED}}_{\mathrm{as}}}$ in nonexpansion states and $\gamma_{1}$*$\overset{‐}{{\mathrm{UNINSURED}}_{\mathrm{as}}}+\gamma_{3}*\overset{‐}{{\mathrm{UNINSURED}}_{\mathrm{as}}}$ in expansion states.

While estimates based on Equation (1) provide average effects over the 2014‐2018 time period, we are primarily interested in how the effects varied over time across these five years, especially in 2017 and 2018. In order to analyze changes over time, we estimate event study models as our preferred set of specifications, where we replace ${\mathrm{POST}}_{t}$ with a set of year dummies. The event study DDD model is

(2) $$y_{\mathrm{iast}}=\varphi +\sum_{t=1}^{T}\theta_{t}\left({\mathrm{UNINSURED}}_{\mathrm{as}}*Y_{t}\right)+\sum_{t=1}^{T}\alpha_{t}{(\mathrm{MEDICAID}}_{s}*Y_{t})+\sum_{t=1}^{T}\beta_{t}\left({\mathrm{UNINSURED}}_{\mathrm{as}}*{\mathrm{MEDICAID}}_{s}*Y_{t}\right)+{\delta X}_{\mathrm{iast}}+\alpha_{\mathrm{as}}+\varepsilon_{\mathrm{iast}}$$

where *Y_t_*, is an indicator for whether year *t* is 2011, 2012, …, 2018, respectively, for *t* = 1, 2,…,7, with 2013 being the reference year and the other terms being as described in Equation (1). Here, the effects of the ACA without the Medicaid expansion during 2014, 2015, …, 2018 are given by $\theta_{3}*{\mathrm{UNINSURED}}_{\mathrm{as}},\theta_{4}*{\mathrm{UNINSURED}}_{\mathrm{as}},$…, $\theta_{7}*{\mathrm{UNINSURED}}_{\mathrm{as}}$, respectively, while the effects of the Medicaid expansion in 2014, 2015, …, 2018 are similarly given by $\beta_{3}*{\mathrm{UNINSURED}}_{\mathrm{as}},\beta_{4}*{\mathrm{UNINSURED}}_{\mathrm{as}},$…$\beta_{7}*{\mathrm{UNINSURED}}_{\mathrm{as}}$.

This event study model also allows us to test the identifying assumptions from our main DDD specification.^11^, ^25^ The first assumption is that, in the absence of the ACA, any changes in the outcomes that would have occurred in 2014‐2018 would not have been systematically correlated with local area uninsured rates, conditional on the controls. Second, differential changes in the outcomes in 2014‐2018 between expansion and nonexpansion states would not have been correlated with prereform uninsured rates. If the event study suggests evidence that changes in the outcomes from 2011 to 2013 are correlated with pre‐ACA uninsured rates (ie, $\theta_{1}$ or $\theta_{2}$ are significant) or the interaction of the local area uninsured rate with Medicaid expansion status (ie, $\beta_{1}$ or $\beta_{2}$ are significant), this would suggest problems with these assumptions.

## 3 RESULTS

Tables 2 and 3 display the implied effects of the ACA based on the coefficient estimates from Equations (1) and (2) multiplied by the 2013 average pretreatment uninsured rate. In the top panel, we display the results from the event study analysis on the year‐by‐year effects of the ACA based on Equation (2), providing the implied effects of the national components of the ACA alone (which is the effect we would expect to see in nonexpansion states) and the fully implemented ACA (which also includes the effect of the Medicaid expansion and is what we would expect to see in expansion states). In the bottom panel, we report the same implied effects over the combined 2014‐2018 postperiod based on Equation (1). In Appendix Table S4 and S5, we discuss alternative specifications to Equation (1) and the robustness of our findings.

**TABLE 2:** Effects of ACA at mean pretreatment uninsured rate on health care access

	Insurance coverage	Primary care doctor	Checkup	Cost barrier
Event Study Model				
ACA without Medicaid Expansion in 2014	0.038*** (0.009)	0.023** (0.009)	‐0.003 (0.010)	‐0.025* (0.012)
ACA without Medicaid Expansion in 2015	0.061*** (0.014)	0.028 (0.025)	‐0.006 (0.019)	‐0.020 (0.010)
ACA without Medicaid Expansion in 2016	0.082*** (0.008)	0.030** (0.009)	0.014 (0.017)	‐0.038** (0.014)
ACA without Medicaid Expansion in 2017	0.077*** (0.015)	0.024* (0.011)	‐0.016 (0.026)	‐0.036 (0.024)
ACA without Medicaid Expansion in 2018	0.076*** (0.016)	0.031** (0.010)	0.008 (0.021)	‐0.040*** (0.008)
ACA with Medicaid Expansion in 2014	0.066*** (0.011)	0.028* (0.014)	0.009 (0.011)	‐0.029*** (0.008)
ACA with Medicaid Expansion in 2015	0.097*** (0.012)	0.047*** (0.013)	0.006 (0.012)	‐0.047*** (0.011)
ACA with Medicaid Expansion in 2016	0.116*** (0.013)	0.051*** (0.011)	0.021 (0.016)	‐0.055*** (0.012)
ACA with Medicaid Expansion in 2017	0.125*** (0.014)	0.051** (0.016)	0.016 (0.021)	‐0.038* (0.016)^^
ACA with Medicaid Expansion in 2018	0.118*** (0.012)	0.063*** (0.015)	0.038 (0.019)	‐0.056*** (0.011)
DDD Model				
ACA without Medicaid Expansion 2014‐2018	0.067*** (0.007)	0.033*** (0.006)	0.024** (0.010)	‐0.034*** (0.007)
ACA with Medicaid Expansion 2014‐2018	0.106*** (0.011)	0.043*** (0.009)	0.040*** (0.012)	‐0.045*** (0.007)
Sample size	2,035,809	2,034,073	2,034,758	2,035,820

Note

Standard errors, heteroscedasticity‐robust and clustered by state, are in parentheses. *** indicates statistically significant at 0.1 percent level; ** 1 percent level; * 5 percent level. BRFSS sampling weights are used. All regressions include state*location type and year*location type fixed effects as well as the controls. In addition, we denote statistically significantly different effect in 2017 and 2018 relative to 2016 by ^^^ at 1 percent level; ^^ at 5 percent level. Each column represents the results from a different regression. Each reported effect estimate represents results from a regression coefficient multiplied by 20.6 percent—the mean uninsured rate in 2013, the year prior to the implementation of the major components of the ACA. To give an example of the interpretation, the first effect in column 1 suggests that the ACA without the Medicaid expansion (ie, the national components of the ACA) led to a 3.8 percentage point increase in the likelihood of reporting any insurance coverage in 2014

**TABLE 3:** Effects of ACA at mean pretreatment uninsured rate on self‐assessed health

	Good or better health	Very good or excellent health	Excellent health	Days not in good physical health	Days not in good mental health	Days with health‐related limitations
Event Study Model						
ACA without Medicaid Expansion in 2014	−0.006 (0.008)	0.018* (0.007)	0.013 (0.009)	−0.114 (0.240)	−0.077 (0.176)	−0.046 (0.126)
ACA without Medicaid Expansion in 2015	0.001 (0.007)	0.020 (0.015)	0.009 (0.009)	0.078 (0.182)	0.270 (0.137)	0.108 (0.159)
ACA without Medicaid Expansion in 2016	0.016** (0.005)	0.042*** (0.009)	0.031*** (0.008)	−0.431 (0.269)	−0.258 (0.177)	−0.217* (0.096)
ACA without Medicaid Expansion in 2017	0.004 (0.013)	0.015 (0.018)	0.001 (0.013)	−0.117 (0.390)	0.094 (0.222)^^^	0.091 (0.248)
ACA without Medicaid Expansion in 2018	0.011 (0.008)	0.035*** (0.009)	0.020* (0.010)	−0.065 (0.365)^^^	−0.343* (0.169)	−0.070 (0.180)
ACA with Medicaid Expansion in 2014	−0.002 (0.008)	0.004 (0.011)	0.007 (0.007)	0.023 (0.166)	−0.310* (0.125)	0.094 (0.189)
ACA with Medicaid Expansion in 2015	−0.005 (0.008)	0.005 (0.013)	0.015 (0.009)	0.061 (0.160)	0.102 (0.151)	0.267 (0.171)
ACA with Medicaid Expansion in 2016	−0.009 (0.008)	0.012 (0.014)	0.024* (0.010)	0.085 (0.184)	−0.176 (0.155)	0.214 (0.153)
ACA with Medicaid Expansion in 2017	−0.005 (0.008)	0.013 (0.011)	0.013 (0.007)	0.008 (0.161)	0.42 (0.216)	0.131 (0.159)
ACA with Medicaid Expansion in 2018	−0.006 (0.008)	0.023 (0.014)	0.032*** (0.009)	0.360 (0.233)	−0.012 (0.134)	0.288 (0.167)
DDD Model						
ACA without Medicaid Expansion 2014‐2018	−0.001 (0.005)	0.011* (0.005)	0.012* (0.006)	−0.197 (0.112)	−0.224 (0.130)	−0.154* (0.077)
ACA with Medicaid Expansion 2014‐2018	−0.002 (0.005)	0.006 (0.008)	0.013* (0.006)	0.001 (0.087)	−0.080 (0.120)	0.127 (0.093)
Sample size	2,035,781	2,035,781	2,035,781	2,016,842	2,018,576	2,027,029

Notes

Standard errors, heteroscedasticity‐robust and clustered by state, are in parentheses. *** indicates statistically significant at 0.1 percent level; ** 1 percent level; * 5 percent level. BRFSS sampling weights are used. All regressions include state*location type and year*location type fixed effects as well as the controls. In addition, we denote statistically significantly different effect in 2017 and 2018 relative to 2016 by ^^^ at 1 percent level; ^^ at 5 percent level. Each column represents the results from a different regression. Each reported effect estimate represents results from a regression coefficient multiplied by 20.6 percent—the mean uninsured rate in 2013, the year prior to the implementation of the major components of the ACA. To give an example of the interpretation, the first effect in column 1 suggests that the ACA without the Medicaid expansion (ie, the national components of the ACA) led to a 0.6 percentage point reduction in the likelihood of reporting good or better health in 2014.

### 3.1 Effects on access

The top panel of Table 2 suggests that, relative to 2013, in states that did not expand Medicaid, the national components of the ACA led to gains in insurance coverage of 3.8 percentage points in 2014, 6.1 in 2015, 8.2 in 2016, 7.7 in 2017, and 7.6 in 2018. In expansion states, the fully implemented ACA led to coverage gains of 6.6 percentage points in 2014, 9.7 in 2015, 11.6 in 2016, 12.5 in 2017, and 11.8 in 2018. Thus, we find no evidence that the administrative changes and political debate surrounding the ACA during 2017 and 2018 led to significantly smaller coverage impacts as compared to 2016.

Our event study model also suggests that the ACA led to increases in the year‐by‐year likelihood of having a primary care doctor. For example, the fully implemented ACA led to a 2.8 percentage point increase in the likelihood of having a primary care doctor in 2014 and a 6.3 percentage point increase in 2018. These results are statistically significant in each year for our estimates of the fully implemented ACA and significant in almost all years for our estimates of the national components. We see a similar pattern in terms of reductions in reporting cost being a barrier to seeking care. With respect to the likelihood of having a checkup in the past year, we also see some growth over time though the estimates in the event study model are not statistically significant in any year and the 2017 and 2018 estimates are not statistically significantly different from 2016.

However, as reported in the bottom panel of Table 2, if we collapse each postreform year into a single postreform time period, we see that the fully implemented ACA led to a statistically significant 4.0 percentage point increase in the likelihood of having a checkup in the postperiod. The effects on insurance coverage and primary care access reported in the bottom panel of Table 2 over the combined 2014‐2018 postperiod are somewhat larger in size (by around 1 percentage point) than those reported in a recent paper using only post‐ACA BRFSS data from 2014 through 2016.^28^

### 3.2 Effects on health

Similarly to previous work considering effects through 2016, our results reported in the top panel of Table 3 suggest that the emergence of an impact on the likelihood of having excellent self‐assessed health appears particularly gradual.^28^ The effect of the fully implemented ACA in expansion states was small and insignificant in 2014, 1.5 percentage points in 2015, 2.4 in 2016, 1.3 in 2017, and 3.2 in 2018. Thus, we do see a smaller increase in 2017 as compared to 2016 and we also see a larger increase occurring in 2018, though none of these effects are statistically different from 2016. We also observe statistically increases in the likelihood of reporting excellent health in some years (2016 and 2018) and very good or excellent health in some years (2014, 2016, and 2018) due to the national components of the ACA.

The bottom panel of Table 3 reports the effects of the ACA on the health outcomes over the combined 2014‐2018 postreform period. For the binary outcomes, nonexpansion states saw a statistically insignificant change in reporting good or better health but a significant 1.1 percentage point increase in very good or excellent health and a 1.2 percentage point increase in excellent health. In expansion states, we observe no statistically significant effect on reporting good or better health and very good or excellent health; however, the probability of reporting excellent health increased by a significant 1.3 percentage points. Finally, while we observe only 3 out of 30 statistically significant effects on the counts of days measures in our event study analysis, we do see a statistically significant reduction in the number of days with health‐related limitations (−0.154 days) in nonexpansion states during the combined 2014‐2018 postperiod.

### 3.3 Testing identifying assumptions

Appendix Tables S2 and S3 present the event study results for the pre‐ACA coefficients associated with our access to care and self‐reported health regressions, respectively. In total, the event study regressions provide 40 coefficients in the pretreatment period (four coefficients in the pre‐ACA period for each of the ten outcomes). We observe only two statistically significant pre‐ACA coefficients out of 40, or five percent, which is exactly what we would expect by chance with a 5 percent rejection rate. Both of the failures are for the checkup variable, suggesting that the results for that outcome should be interpreted with caution.

### 3.4 Specification checks

The results of our many specification checks are described in detail in the Appendix. These include dropping those in the catch‐all cell phone “area type,” excluding 19‐ to 25‐year‐olds since they may have been already treated by the ACA‐dependent coverage expansion, dropping early and late expanding states in order to better isolate the impact of the Medicaid expansion,^11^, ^47^ and using state rather than local‐area‐type baseline uninsured rates with and without additional state‐level controls for economic and labor market conditions. In addition, we explore the sensitivity of our results to treating the coefficient on the ${\mathrm{MEDICAID}}_{\mathrm{st}}*{\mathrm{POST}}_{t}$ term as part of the causal effect of the Medicaid expansion. Appendix Tables S4 and S5 report the results of these specification checks. Taken as a whole, these estimates are broadly consistent with our baseline results.

## 4 DISCUSSION

In this paper, we examine the impact of the ACA on access to care and self‐assessed health during the first two years of the Trump administration (2017‐2018). The addition of 2017 and 2018 data allows us to examine whether gains in access and health continued beyond what was previously documented in the published literature using data through 2016. One reason why we might expect to see changes in trends after 2016 is the transition of political party in the White House in 2017, which led to a policy shift and several changes in the administration of the ACA (such as reductions in outreach funding and the duration of open enrollment as well as the discontinuation of CSR payments), as well as a near repeal in the summer of 2017.

Relative to 2013, our results suggest that insurance coverage in Medicaid expansion states increased by 6.6 percentage points in 2014, 9.7 in 2015, 11.6 in 2016, 12.5 in 2017, and 11.8 in 2018. In states that did not expand Medicaid, gains in insurance coverage were 3.8 percentage points in 2014, 6.1 in 2015, 8.2 in 2016, 7.7 in 2017, and 7.6 in 2018. Thus, we find no evidence that the administrative changes and political debate surrounding the ACA during 2017 and 2018 led to significantly smaller coverage increases as compared to 2016. Given that many of these administrative changes focus on Marketplace coverage, it is interesting to note that the reported gains are only modestly larger in Medicaid expansion states than in nonexpansion states, implying that these persistent insurance gains are mostly attributable to the national components of the ACA, including subsidized Marketplace coverage.^28^

Another study^12^ examining the relative coverage impact of the Medicaid expansion versus the other components of the ACA found that 60 percent of the ACA coverage gains after two years (2014‐2015) can be causally attributed to the Medicaid expansion. While our results suggest closer to a 40 percent share attributable to the Medicaid expansion after five years, a 54 percent share sits within the 95 percent confidence interval for that point estimate. This implies we cannot rule out a 54 percent coverage share coming from the Medicaid expansion. Thus, given differences in data, samples, time frames, and methods used in other work, we view our findings as broadly consistent with estimates from the literature.

Similarly to previous work considering effects through 2016, our results regarding self‐assessed health suggest that the emergence of an impact on the likelihood of having excellent health was particularly gradual, with the effect of the fully implemented ACA in expansion states being small and insignificant in 2014, 1.5 percentage points in 2015, 2.4 in 2016, 1.3 in 2017, and 3.2 in 2018.^28^ The smaller increase in 2017 and the larger increase occurring in 2018 are each not statistically different from the 2016 estimate. Thus, we do not observe an impact of administrative changes and the general debate surrounding the ACA on the trend in reporting increased gains in excellent self‐assessed health.

The literature has documented multiple potential reasons why the national components of the ACA might be more effective than the Medicaid expansion at improving access and self‐assessed health.^28^ For example, more generous reimbursement rates in Marketplace plans may make it easier for Marketplace enrollees to find a primary care doctor that accepts their policy as compared to Medicaid enrollees, despite growing evidence that Marketplace plans provide narrow networks.^48^, ^49^, ^50^, ^51^, ^52^ In addition, it has been documented that Marketplace enrollees are more health literate than those gaining coverage through prior expansions, a group that may be more representative of those gaining coverage through the ACA Medicaid expansions.^53^ Thus, Marketplace enrollees may be better initially positioned to make the most of their newly gained coverage.

Debate about the future of the ACA, a major driver of health, continues to be a dominant theme in both national and state politics. Several presidential candidates expressed support for different versions of “Medicare for all,” while maintaining state Medicaid expansion status appeared to play a big role in recent gubernatorial elections in such states as Kentucky (that expanded Medicaid) and Mississippi (which did not). This ongoing debate, as well as the fact that multiple states have (Virginia and Maine) or are planning to (Idaho, Nebraska, and Utah) expand their Medicaid program subsequent to 2018, suggests the need for future work that continues to examine the evolving impact of the ACA on access to care and self‐assessed health, in addition to the other key outcomes that have been featured in the ACA literature.^2^ While the changes to the ACA we examined did not lead to short‐run reductions in access to care or changes in the trend in reporting increased gains in excellent self‐assessed health in 2017 and 2018, that does not tell us whether the impact of these changes will differ in the long run or how the other potential changes described above will impact these outcomes.

## Supporting information

Appendix S1

Appendix S2
