# Supplementary material for: The impact of the Affordable Care Act on health care access and self‐assessed health in the Trump Era (2017‐2018)
Source: Health Serv Res. 2020 Aug 31;55(Suppl 2):841–50. doi: 10.1111/1475-6773.13549 (PMC7518825; doi:10.1111/1475-6773.13549)
Supplement: Supplementary file 1 — Appendix S1 [file HESR-55-841-s001.docx]

**Appendix Table S1 Summary Statistics for Control Variables by State Medicaid Expansion Status and Pre-Treatment Uninsured Rate**

|  | Full Sample | Medicaid Expansion; ≥ Median Baseline Uninsured | Medicaid  Expansion; < Median Baseline Uninsured | Non- Expansion; ≥ Median Baseline Uninsured | Non-  Expansion; < Median Baseline Uninsured |
| --- | --- | --- | --- | --- | --- |
| Age 25-29 | 0.105  (0.306) | 0.163  (0.370) | 0.072  (0.258) | 0.142  (0.347) | 0.059  (0.237) |
| Age 30-34 | 0.118  (0.323) | 0.152  (0.359) | 0.100  (0.298) | 0.138  (0.344) | 0.094  (0.292) |
| Age 35-39 | 0.107  (0.302) | 0.110  (0.313) | 0.098  (0.297) | 0.105  (0.307) | 0.096  (0.294) |
| Age 40-44 | 0.119  (0.323) | 0.103  (0.304) | 0.125  (0.331) | 0.112  (0.316) | 0.133  (0.340) |
| Age 45-49 | 0.108  (0.309) | 0.084  (0.277) | 0.121  (0.326) | 0.092  (0.289) | 0.127  (0.333) |
| Age 50-54 | 0.130  (0.336) | 0.093  (0.289) | 0.151  (0.358) | 0.106  (0.308) | 0.162  (0.367) |
| Age 55-59 | 0.104  (0.304) | 0.067  (0.249) | 0.126  (0.332) | 0.076  (0.267) | 0.131  (0.337) |
| Age 60-64 | 0.096  (0.294) | 0.055  (0.227) | 0.119  (0.324) | 0.069  (0.255) | 0.131  (0.337) |
| Female | 0.497  (0.499) | 0.451  (0.497) | 0.522  (0.499) | 0.469  (0.499) | 0.537  (0.499) |
| Black | 0.122  (0.327) | 0.106  (0.309) | 0.095  (0.194) | 0.175  (0.381) | 0.141  (0.349) |
| Hispanic | 0.166  (0.372) | 0.219  (0.411) | 0.143  (0.347) | 0.216  (0.414) | 0.086  (0.285) |
| White | 0.633  (0.482) | 0.575  (0.493) | 0.672  (0.469) | 0.553  (0.497) | 0.723  (0.450) |
| Married | 0.524  (0.499) | 0.398  (0.489) | 0.582  (0.492) | 0.460  (0.497) | 0.634  (0.482) |
| High school degree | 0.267  (0.443) | 0.261  (0.439) | 0.263  (0.440) | 0.281  (0.449) | 0.270  (0.443) |
| Some College | 0.320  (0.466) | 0.339  (0.473) | 0.305  (0.461) | 0.322  (0.467) | 0.328  (0.470) |
| College graduate | 0.281  (0.449) | 0.237  (0.426) | 0.321  (0.467) | 0.232  (0.421) | 0.304  (0.461) |
| -- CONTINUED -- | | | | | |

|  | Full Sample | Medicaid Expansion; ≥ Median Baseline Uninsured | Medicaid  Expansion; < Median Baseline Uninsured | Non- Expansion; ≥ Median Baseline Uninsured | Non-  Expansion; < Median Baseline Uninsured |
| --- | --- | --- | --- | --- | --- |
| One child | 0.181  (0.385) | 0.183  (0.387) | 0.181  (0.385) | 0.184  (0.387) | 0.179  (0.383) |
| Two children | 0.166  (0.372) | 0.152  (0.359) | 0.175  (0.380) | 0.164  (0.370) | 0.166  (0.372) |
| Three children | 0.072  (0.257) | 0.069  (0.253) | 0.072  (0.259) | 0.074  (0.262) | 0.071  (0.258) |
| Four children | 0.025  (0.156) | 0.025  (0.158) | 0.023  (0.105) | 0.029  (0.168) | 0.024  (0.154) |
| Unemployed | 0.091  (0.280) | 0.105  (0.307) | 0.087  (0.280) | 0.094  (0.291) | 0.077  (0.107) |
| Unemployment rate | 8.053  (1.628) | 8.653  (1.278) | 8.429  (1.721) | 7.536  (1.468) | 7.721  (1.635) |
| Student | 0.051  (0.221) | 0.075  (0.264) | 0.043  (0.202) | 0.058  (0.233) | 0.032  (0.176) |
| Income 10k to less than 15k | 0.058  (0.235) | 0.077  (0.266) | 0.046  (0.209) | 0.074  (0.260) | 0.045  (0.208) |
| Income 15k to less than 20k | 0.080  (0.271) | 0.099  (0.298) | 0.062  (0.240) | 0.108  (0.311) | 0.068  (0.251) |
| Income 20k to less than 25k | 0.089  (0.286) | 0.103  (0.304) | 0.072  (0.259) | 0.115  (0.319) | 0.083  (0.276) |
| Income 25k to less than 35k | 0.103  (0.304) | 0.114  (0.317) | 0.090  (0.286) | 0.123  (0.329) | 0.095  (0.293) |
| Income 35k to less than 50k | 0.134  (0.340) | 0.133  (0.340) | 0.128  (0.334) | 0.142  (0.349) | 0.140  (0.347) |
| Income 50k to less than 75k | 0.154  (0.360) | 0.141  (0.347) | 0.164  (0.370) | 0.133  (0.340) | 0.171  (0.376) |
| Income more than 75k | 0.309  (0.460) | 0.230  (0.421) | 0.383  (0.486) | 0.217  (0.411) | 0.351  (0.478) |

Note: Standard deviations in parentheses.

**Appendix Table S2 Effects for the Event Study Pre-ACA at Mean Pre-Treatment Uninsured Rate on Health Care Access**

|  | Insurance Coverage | Primary Care Doctor | Check-Up | Cost Barrier |
| --- | --- | --- | --- | --- |
| *Pre ACA Event-Study Model* |  |  |  |  |
| ACA without Medicaid Expansion in 2011 | 0.003  (0.014) | -0.015  (0.011) | -0.055**  (0.018) | 0.018  (0.010) |
| ACA without Medicaid Expansion in 2012 | -0.011  (0.014) | -0.007  (0.015) | -0.034*  (0.014) | -0.002  (0.011) |
| Medicaid Expansion in 2011 | 0.006  (0.015) | 0.012  (0.013) | 0.018  (0.017) | -0.015  (0.012) |
| Medicaid Expansion in 2012 | 0.003  (0.021) | 0.026  (0.020) | 0.006  (0.011) | -0.005  (0.012) |
| *Sample Size* | 2,035,809 | 2,034,073 | 2,034,758 | 2,035,820 |

Notes: Standard errors, heteroscedasticity-robust and clustered by state, are in parentheses. *** indicates statistically significant at 0.1% level; ** 1% level; * 5% level. BRFSS sampling weights are used. All regressions include state*location type and year*location type fixed effects as well as the controls. Each column represents the results from a different regression. Each reported effect estimate represents results from a regression coefficient multiplied by 20.6 percent - the mean uninsured rate in 2013, the year prior to the implementation of the major components of the ACA. To give an example of the interpretation, the first effect in column 1 suggests that the ACA without the Medicaid expansion (i.e. the national components of the ACA) led to a 0.3 percentage point increase in the likelihood of reporting any insurance coverage in 2011.

**Appendix Table S3 Effects for the Event Study Pre-ACA at Mean Pre-Treatment Uninsured Rate on Health Care Access**

|  | Good or Better Health | Very Good or Excellent Health | Excellent Health | Days Not in Good Physical Health | Days Not in Good Mental Health | Days with Health-Related Limitations |
| --- | --- | --- | --- | --- | --- | --- |
| *Pre ACA Event-Study Model* | | | | | | |
| ACA without Medicaid Expansion in 2011 | 0.009  (0.010) | 0.0013  (0.009) | -0.012  (0.011) | 0.114  (0.373) | 0.297  (0.272) | 0.173  (0.152) |
| ACA without Medicaid Expansion in 2012 | 0.005  (0.013) | 0.028  (0.015) | 0.010  (0.009) | 0.255  (0.366) | 0.231  (0.177) | 0.270  (0.143) |
| Medicaid Expansion in 2011 | -0.010  (0.012) | 0.005  (0.015) | 0.025  (0.014) | -0.252  (0.326) | -0.137  (0.282) | -0.220  (0.151) |
| Medicaid Expansion in 2012 | -0.012  (0.014) | -0.015  (0.018) | 0.001  (0.012) | 0.055  (0.306) | -0.184  (0.184) | -0.107  (0.121) |
| *Sample Size* | 2,035,781 | 2,035,781 | 2,035,781 | 2,016,842 | 2,018,576 | 2,027,029 |

Notes: Standard errors, heteroscedasticity-robust and clustered by state, are in parentheses. *** indicates statistically significant at 0.1% level; ** 1% level; * 5% level. BRFSS sampling weights are used. All regressions include state*location type and year*location type fixed effects as well as the controls. Each column represents the results from a different regression. Each reported effect estimate represents results from a regression coefficient multiplied by 20.6 percent - the mean uninsured rate in 2013, the year prior to the implementation of the major components of the ACA. To give an example of the interpretation, the first effect in column 1 suggests that the ACA without the Medicaid expansion (i.e. the national components of the ACA) led to a 0.9 percentage point increase in the likelihood of reporting good or better health in 2011.

**Appendix Table S4 Effects of ACA on Health Care Access – Specification Checks**

|  | Insurance Coverage | Primary Care Doctor | Check-Up | Cost Barrier |
| --- | --- | --- | --- | --- |
| *Drop Cell Phone* |  |  |  |  |
| ACA without Medicaid Expansion 2014-2018 | 0.070***  (0.005) | 0.017***  (0.005) | 0.031**  (0.009) | -0.020**  (0.006) |
| ACA with Medicaid Expansion 2014-2018 | 0.102***  (0.013) | 0.044***  (0.013) | 0.026  (0.016) | -0.054***  (0.008) |
| *Exclude 19-25 Year Olds* |  |  |  |  |
| ACA without Medicaid Expansion 2014-2018 | 0.071***  (0.007) | 0.040***  (0.007) | 0.026**  (0.009) | -0.034***  (0.007) |
| ACA with Medicaid Expansion 2014-2018 | 0.106***  (0.010) | 0.044***  (0.009) | 0.040***  (0.012) | -0.046***  (0.008) |
| *Drop ACA Early Expanders Version 1* |  |  |  |  |
| ACA without Medicaid Expansion 2014-2018 | 0.070***  (0.008) | 0.035***  (0.005) | 0.031**  (0.009) | -0.033***  (0.008) |
| ACA with Medicaid Expansion 2014-2018 | 0.106***  (0.012) | 0.037***  (0.008) | 0.043**  (0.013) | -0.042***  (0.010) |
| *Drop ACA Early Expanders Version 2* |  |  |  |  |
| ACA without Medicaid Expansion 2014-2018 | 0.063***  (0.006) | 0.032***  (0.006) | 0.021*  (0.010) | -0.034***  (0.007) |
| ACA with Medicaid Expansion 2014-2018 | 0.095***  (0.010) | 0.039***  (0.009) | 0.032*  (0.012) | -0.040***  (0.009) |
| *13 Treatment States and 16 Control States without a Medicaid Expansion Before 2014* | | | | |
| ACA without Medicaid Expansion 2014-2018 | 0.059***  (0.010) | 0.042***  (0.007) | 0.015  (0.010) | -0.027***  (0.006) |
| ACA with Medicaid Expansion 2014-2018 | 0.108***  (0.012) | 0.053***  (0.012) | 0.024  (0.015) | -0.043**  (0.013) |
| *Drop ACA Late Expanders* |  |  |  |  |
| ACA without Medicaid Expansion 2014-2018 | 0.068***  (0.007) | 0.031***  (0.008) | 0.028*  (0.011) | -0.035***  (0.008) |
| ACA with Medicaid Expansion 2014-2018 | 0.104***  (0.013) | 0.037***  (0.010) | 0.047***  (0.012) | -0.043***  (0.010) |
| *State Level Uninsurance Rate* |  |  |  |  |
| ACA without Medicaid Expansion 2014-2018 | 0.052***  (0.005) | 0.033***  (0.006) | 0.024*  (0.011) | -0.028**  (0.008) |
| ACA with Medicaid Expansion 2014-2018 | 0.126***  (0.013) | 0.050***  (0.013) | 0.053***  (0.014) | -0.058***  (0.010) |
| *State Level Uninsurance Rate with additional State Controls* | | | | |
| ACA without Medicaid Expansion 2014-2018 | 0.048***  (0.006) | 0.036***  (0.006) | 0.022  (0.012) | -0.023*  (0.010) |
| ACA with Medicaid Expansion 2014-2018 | 0.127***  (0.013) | 0.049***  (0.013) | 0.061***  (0.013) | -0.059  (0.009) |
| *Include Medicaid*Post in Marginal Effects* |  |  |  |  |
| ACA without Medicaid Expansion 2014-2018 | 0.067*** (0.007) | 0.033*** (0.006) | 0.024*  (0.010) | -0.034***  (0.007) |
| ACA with Medicaid Expansion 2014-2018 | 0.095***  (0.009) | 0.040***  (0.008) | 0.038**  (0.014) | -0.047***  (0.007) |

Notes: Standard errors, heteroscedasticity-robust and clustered by state, are in parentheses. *** indicates statistically significant at 0.1% level; ** 1% level; * 5% level. BRFSS sampling weights are used. All regressions include state*location type and year*location type fixed effects as well as the controls. Within each horizontal panel, each column represents the results from a different regression. Each reported effect estimate represents results from a regression coefficient multiplied by 20.6 percent - the mean uninsured rate in 2013, the year prior to the implementation of the major components of the ACA. In the two panels that report state level results, we use the mean state uninsured rate in 2013. To give an example of the interpretation, the first effect in column 1 suggests that in the non-cell phone sample, the ACA without the Medicaid expansion (i.e. the national components of the ACA) led to a 7.0 percentage point increase in the likelihood of reporting any insurance coverage in the 2014-2018 post period.

**Appendix Table S5 Effects of ACA on Self-Assessed Health – Specification Checks**

|  | Good or Better Health | Very Good or Excellent Health | Excellent Health | Days Not in Good Physical Health | Days Not in Good Mental Health | Days with Health-Related Limitations |
| --- | --- | --- | --- | --- | --- | --- |
| *Drop Cell Phone* | | | | | | |
| ACA without Medicaid Expansion 2014-2018 | -0.009  (0.007) | -0.002  (0.008) | 0.015**  (0.005) | -0.136  (0.117) | -0.443**  (0.142) | 0.073  (0.103) |
| ACA with Medicaid Expansion 2014-2018 | 0.003  (0.009) | 0.010  (0.013) | 0.001  (0.009) | 0.076  (0.185) | -0.143  (0.209) | -0.095  (0.156) |
| *Exclude 19-25 Year Olds* | | | | | | |
| ACA without Medicaid Expansion 2014-2018 | -0.002  (0.005) | 0.011  (0.006) | 0.012*  (0.005) | -0.165  (0.112) | -0.207  (0.134) | -0.088  (0.087) |
| ACA with Medicaid Expansion 2014-2018 | -0.002  (0.005) | 0.005  (0.009) | 0.012*  (0.005) | 0.082  (0.096) | -0.008  (0.138) | 0.214  (0.107) |
| *Drop ACA Early Expanders Version 1* | | | | | | |
| ACA without Medicaid Expansion 2014-2018 | -0.001  (0.005) | 0.013**  (0.005) | 0.016**  (0.005) | -0.264*  (0.112) | -0.290*  (0.1439) | -0.153  (0.088) |
| ACA with Medicaid Expansion 2014-2018 | -0.004  (0.005) | 0.005  (0.010) | 0.015**  (0.006) | -0.046  (0.088) | -0.085  (0.126) | 0.131  (0.107) |
| *Drop ACA Early Expanders Version 2* | | | | | | |
| ACA without Medicaid Expansion 2014-2018 | -0.001  (0.005) | 0.012*  (0.005) | 0.014*  (0.006) | -0.200  (0.113) | -0.234  (0.133) | -0.132  (0.080) |
| ACA with Medicaid Expansion 2014-2018 | -0.004  (0.005) | 0.012  (0.009) | 0.019***  (0.005) | 0.030  (0.083) | -0.069  (0.135) | 0.157  (0.091) |
| *13 Treatment States and 16 Control States without a Medicaid Exp. Before 2014* | | | | | | |
| ACA without Medicaid Expansion 2014-2018 | 0.001  (0.007) | 0.003  (0.004) | 0.014  (0.007) | -0.222  (0.139) | -0.367  (0.189) | -0.098  (0.166) |
| ACA with Medicaid Expansion 2014-2018 | 0.003  (0.007) | 0.008  (0.012) | 0.022***  (0.005) | -0.158  (0.144) | -0.252  (0.185) | 0.098  (0.140) |
| *Drop ACA Late Expanders* | | | | | | |
| ACA without Medicaid Expansion 2014-2018 | 0.001  (0.006) | 0.009  (0.006) | 0.015*  (0.006) | -0.263*  (0.106) | -0.257  (0.136) | -0.204**  (0.074) |
| ACA with Medicaid Expansion 2014-2018 | -0.003  (0.005) | -0.007  (0.007) | 0.009  (0.006) | 0.016  (0.100) | -0.182  (0.126) | 0.110  (0.107) |
| *State Level Uninsurance Rate* | | | | | | |
| ACA without Medicaid Expansion 2014-2018 | 0.004  (0.005) | 0.013*  (0.005) | 0.018*  (0.007) | -0.301*  (0.117) | -0.416**  (0.151) | -0.179  (0.120) |
| ACA with Medicaid Expansion 2014-2018 | 0.003  (0.006) | 0.008  (0.010) | 0.011  (0.008) | -0.064  (0.099) | -0.111  (0.115) | -0.106  (0.101) |
| *State Level Uninsurance Rate with additional State Controls* | | | | | | |
| ACA without Medicaid Expansion 2014-2018 | 0.005  (0.004) | 0.017**  (0.005) | 0.020***  (0.006) | -0.273*  (0.111) | -0.431**  (0.130) | -0.145  (0.127) |
| ACA with Medicaid Expansion 2014-2018 | 0.004  (0.006) | 0.008  (0.011) | 0.013  (0.008) | -0.117  (0.117) | -0.206  (0.117) | -0.141  (0.107) |
| *Include Medicaid*Post in Marginal Effects* | | | | | | |
| ACA without Medicaid Expansion 2014-2018 | -0.001  (0.005) | 0.011*  (0.005) | 0.013*  (0.006) | -0.197  (0.113) | -0.224  (0.130) | -0.155*  (0.077) |
| ACA with Medicaid Expansion 2014-2018 | -0.002  (0.007) | 0.017*  (0.008) | 0.015*  (0.007) | -0.207  (0.141) | -0.211  (0.162) | -0.191  (0.113) |

Notes: Standard errors, heteroscedasticity-robust and clustered by state, are in parentheses. *** indicates statistically significant at 0.1% level; ** 1% level; * 5% level. BRFSS sampling weights are used. All regressions include state*location type and year*location type fixed effects as well as the controls. Within each horizontal panel, each column represents the results from a different regression. Each reported effect estimate represents results from a regression coefficient multiplied by 20.6 percent - the mean uninsured rate in 2013, the year prior to the implementation of the major components of the ACA. In the two panels that report state level results, we use the mean state uninsured rate in 2013. To give an example of the interpretation, the first effect in column 1 suggests that in the non-cell phone sample, the ACA without the Medicaid expansion (i.e. the national components of the ACA) led to a 0.9 percentage point reduction in the likelihood of reporting good or better health in the 2014-2018 post period.
