# Supplementary material for: The impact of the Affordable Care Act on health care access and self‐assessed health in the Trump Era (2017‐2018)
Source: Health Serv Res. 2020 Aug 31;55(Suppl 2):841–50. doi: 10.1111/1475-6773.13549 (PMC7518825; doi:10.1111/1475-6773.13549)
Supplement: Supplementary file 2 — Appendix S2 [file HESR-55-841-s002.docx]

**Appendix**

**Descriptive Analysis**

Appendix Table S1 contains the summary statistics for the control variables, stratified by area pretreatment uninsured rate and state Medicaid expansion status. Individuals who live in Medicaid expansion states with prereform uninsured rates below the median (column 3) were, on average, more educated, more likely to be employed, and had higher incomes than those in the other groups. Our research design accounts for these differences by including these variables as controls.

**Testing for Differential Pretreatment Trends**

Appendix Tables S2 and S3 present the event study results for the pre-ACA period, where we present the point estimates for the implied effects for only the ACA portion and the Medicaid portion, respectively. Appendix Table S2 reports the implied effects of the ACA on the health care access outcomes, and appendix Table S3 does the same for self-assessed health outcomes. The key finding (beyond the information given in the main text of the paper) is that there is little evidence of problematic pretreatment trends. For all access outcomes (table 2), the pre-ACA implied effects are contained in the confidence interval and include zero. Only the checkup outcome generates two statistically significant pretreatment coefficients associated with the national components of the ACA. For the self-assessed health outcomes, we find no statistically significant pretreatment coefficient.

**Specification Checks**

Appendix Tables S4 and S5 present the results of a series of additional specification checks of our baseline models. Appendix Table S4 presents the results for our access to care outcomes, and appendix Table S5 presents the results for our self-assessed health outcomes. The first two panels check the sensitivity to our sample when we drop the cell phone sample because we do not know their geographic location within a state and exclude 19- to 25-year-olds because they should have been mostly “treated’ with the dependent care coverage mandate portion of the ACA that came into effect at the end of 2010. We observe a reduction in the point estimate for the ACA effect on very good or excellent health, which may highlight heterogeneous effects by type of survey respondent.

In the next four panels, we address concerns of the timing of state Medicaid expansion decisions. The first two of these checks drops early expansion states using two different classifications of such states (California, Connecticut, Washington DC, Minnesota, New Jersey, and Washington in version 1 vs. Delaware, Washington DC, Massachusetts, New Your, and Vermont in version 2) and re-estimates our baseline models. The third restricts the sample to the 13 treatment states and 16 control states that did not have some form of Medicaid expansion prior to January 2014 in order to better isolate the full Medicaid expansion effect. The fourth drops states that expanded after January 2014. In all four models, the results are generally similar to our baseline findings. In the sample of 13 treatment states and 16 control states and dropping the late expanders’ results in a statistically insignificant effect of the ACA alone on very good or excellent health and the effect of the full ACA on excellent health, but this is part because of an increase in the standard error rather than a meaningful change in the point estimate.

Our next two specification checks examine the robustness of our results to a different measure of the local area pretreatment uninsured rate. To do this, we run two alternative specifications where we aggregate the BRFSS uninsurance rate to the state level. In the first of these checks that is the only change we make. In the second, we also add additional controls for labor market and economic conditions at the state level, including the percent of health care jobs out of all jobs, the percent of government jobs out of all jobs, and state per capita GDP. Both sets of results mirror our baseline results.

Finally, we have re-estimated our implied effects in order to include the “Medicaid * Post” term rather than treating it as a confounder. The implied effects of the fully implemented ACA, which includes the Medicaid expansion, are very similar whether we include the “Medicaid*post” term for our access outcomes. The implied effects are somewhat larger when we include this term for the probability of having a primary care doctor, having a checkup, and reporting cost being a barrier to care. Conversely, the implied effect on insurance coverage is actually somewhat smaller when we include this term (a 10.6 percentage point increase vs. a 9.5 percentage point increase). The fully implemented ACA results are also similar for our health outcomes whether we include the “Medicaid*post” term. In each case, the coefficient on the excellent health outcome is statistically significant. In addition, when we include the “Medicaid*post” term the coefficient on the very good or excellent health outcome becomes statistically significant.
